# Site-directed mutagenesis around the CuA site of a polyphenol oxidase from *Coreopsis grandiflora* (*cg*AUS1)

**DOI:** 10.1016/j.febslet.2015.02.009

**Published:** 2015-03-24

**Authors:** Cornelia Kaintz, Rupert L. Mayer, Franz Jirsa, Heidi Halbwirth, Annette Rompel

**Affiliations:** a Universität Wien, Fakultät für Chemie, Institut für Biophysikalische Chemie, Althanstraße 14, 1090 Wien, Austria; b Universität Wien, Department of Analytical Chemistry, Währinger Straße 38, 1090 Vienna, Austria; c Universität Wien, Department of Inorganic Chemistry, Althanstraße 14, 1090 Vienna, Austria; d University of Technology Vienna, Institute of Chemical Engineering, Getreidemarkt 9, 1060 Vienna, Austria

**Keywords:** AAS, atomic absorption spectroscopy, AmAS1, aureusidin synthase from *Antirrhinum majus* (protein), *ao*TYR, tyrosinase from *Aspergillus oryzae* (protein), *bm*TYR, tyrosinase from *Bacillus megaterium* (protein), *cg*AUS1, aurone synthase from *Coreopsis grandiflora* (protein), *cgAUS1*, aurone synthase from *Coreopsis grandiflora* (gene), F-AAS, flame atomic absorption spectroscopy, GF-AAS, graphite furnace atomic absorption spectroscopy, GST, glutathione S-transferase, *ib*CO, catechol oxidase from *Ipomoea batatas* (protein), IPTG, isopropyl-β-d-thiogalactopyranoside, LC, liquid chromatography, LC/MS, liquid chromatography / mass spectrometry, nanoUHPLC–ESI-MS/MS, ultra high performance liquid chromatography–electrospray tandem mass spectrometry, PES, poly(oxy-1,4-phenylsulfonyl-1,4-phenyl), PHC, 2′,3,4,4′,6′-pentahydroxychalcone, PMSF, phenylmethylsulfonylfluorid, PPO, polyphenol oxidase, PPO-6, dandelion PPO-6 from *Taraxacum officinale* (protein), TBC, 4-tert-butylcatechol, THC, 2′,4,4′,6′-tetrahydroxychalcone, *vv*CO, catechol oxidase from *Vitis vinifera* (protein), Type-3 copper protein, Polyphenol oxidase (PPO), Aurone synthase (AUS), Site-directed mutagenesis, 4-Deoxyaurone, Copper binding site

## Abstract

Aurone synthase from *Coreopsis grandiflora* (*cg*AUS1), catalyzing conversion of butein to sulfuretin in a type-3 copper center, is a rare example of a polyphenol oxidase involved in anabolism. Site-directed mutagenesis around the CuA site of AUS1 was performed, and recombinant enzymes were analyzed by mass spectrometry. Replacement of the coordinating CuA histidines with alanine resulted in the presence of a single copper and loss of diphenolase activity. The thioether bridge-building cysteine and a phenylalanine over the CuA site, exchanged to alanine, have no influence on copper content but appear to play an important role in substrate binding.

## Highlights

• Site-directed mutations of AUS1 around the CuA site were generated and verified.
• All mutations led to loss of diphenolase activity with butein as substrate.
• Exchange of histidines in the CuA resulted in enzymes containing only one Cu.
• F273 mutation to alanine did not increase the monophenolase activity.
• C97 mutation eliminated the diphenolase activity, but 2 Cu atoms were incorporated.

## 1 Introduction

**Fig. 1:**
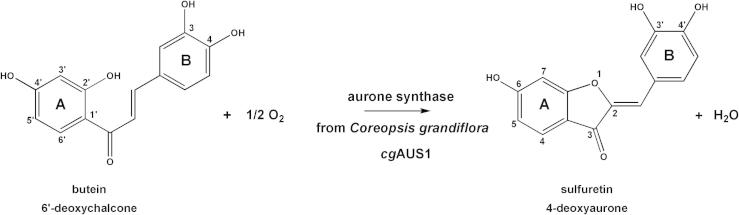
Reaction catalyzed by *cg*AUS1 in the 4-deoxyaurone pathway. Butein (6′-deoxychalcone) is converted to sulfuretin (4-deoxyaurone). Note the differing atom numbering of the chalcone and the aurone.

Type-3 copper enzymes contain two copper atoms (CuA and CuB) and bind dioxygen in a characteristic “side-on” bridging mode (μ-η^2^:η^2^) between both copper atoms [1]. Polyphenol oxidases (PPOs) are an important class of type-3 copper enzymes and are predominantly involved in pigment formation leading to browning of fruits and vegetables. They can also play a role during aurone formation or in the plant pigment biosynthesis [2–6]. The most prominent PPOs are classified by enzyme nomenclature into tyrosinases (monophenol, *o*-diphenol: oxygen oxidoreductase, EC 1.14.18.1), catechol oxidases (CO, *o*-diphenol:oxygen oxidoreductase, EC 1.10.3.1), aureusidin synthase (2′,4,4′,6′-tetrahydroxychalcone 4′-*O*-*β*-d-glucoside:oxygen oxidoreductase, EC 1.21.3.6) and laccases (benzenediol:oxygen oxidoreductase, EC 1.10.3.2) [2]. While tyrosinases catalyze both the hydroxylation of phenols to *o*-diphenols and the subsequent oxidation to their related, highly reactive *o*-quinones, catechol oxidases catalyze only the oxidation reaction of *o*-diphenols to *o*-quinones. Laccases are multicopper oxidase enzymes, which catalyze the oxidation of phenolic and non-phenolic compounds by withdrawing the electron from the substrate and converting them in free radicals, which then polymerize [7]. Aureusidin synthase (AmAS1) and the recently described aurone synthase are special PPOs as they are both involved in aurone formation [5,8]. Aureusidin synthase (Uniprot accession number Q9FRX6) was found in the 4-hydroxyaurone pathway of *Antirrhinum majus* (old Scrophulariaceae, now Plantaginaceae) and *Helichrysum bracteatum* (Asteraceae) and catalyzes two reactions, the hydroxylation and the oxidative cyclization of 2′,3,4,4′,6′-pentahydroxychalcone (PHC) and 2′,4,4′,6′-tetrahydroxychalcone (THC) into aureusidin and bracteatin, respectively [8–10]. The polyphenol oxidase homologue aurone synthase (Uniprot accession number A0A075DN54) in *Coreopsis grandiflora* (*cg*AUS1) is involved in the 4-deoxyaurone pathway and catalyzes only the conversion of chalcones to aurones, by oxidizing butein (6′-deoxychalcone) to sulfuretin (4-deoxyaurone) [5,11]. Thus, unlike aureusidin synthase, which accepts both monohydroxylated and dihydroxylated substrates, aurone synthase accepts only dihydroxylated substrates, with hydroxy groups in positions 3 and 4 of ring B (Fig. 1) [5,11].

**Fig. 2:**
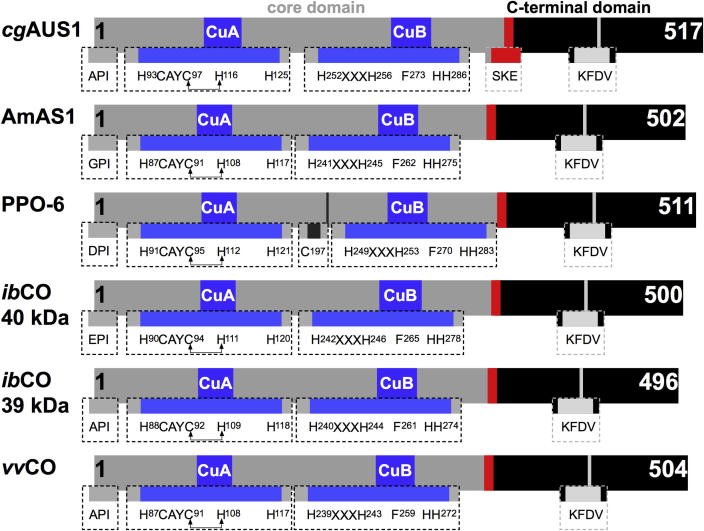
Schematic alignment of plant PPO pro-enzymes (without transit peptide). *cg*AUS1: aurone synthase from *Coreopsis grandiflora* (Uniprot accession number A0A075DN54), AmAS1: aureusidin synthase from *Antirrhinum majus* (Uniprot accession number Q9FRX6), PPO-6: dandelion PPO from *Taraxacum officinale* (Uniprot accession number I7HUF2), *ib*CO 40 kDa: 40 kDa isoform of *Ipomoea batatas* (Uniprot accession number Q9MB14), *ib*CO 39 kDa is the 39 kDa isoform of *Ipomoea batatas* (Uniprot accession number Q9ZP19), *vv*CO: catechol oxidase from *Vitis vinifera* (Uniprot accession number P43311). The grey part is the core domain, the black part the C-terminal domain, the red part is the putative cleavage region.

The total sequence (including the transit peptide) of aurone synthase (*cg*AUS1) contains 602 amino acids, corresponding to a mass of 68 kDa [5]. The pro-enzyme (also called latent enzyme, which contains no transit peptide) starts with the amino acids APITAPDI and consists of 517 amino acids, leading to a mass of 59 kDa (see Fig. 2, *cg*AUS1). Typically the transit peptide of plant PPOs is cleaved in vivo after transport to the target location (in case of AUS1 as in most plant PPOs the lumen of the thylakoid membranes in chloroplast), thus the enzyme is expressed in the latent form [5,12–14]. Proteolytic cleavage of the C-terminal domain results in an active enzyme (core domain), with a mass of 40 kDa [5]. In *cg*AUS1 the putative cleavage region is near the SKE motif, marked in red in Fig. 2. Typical highly conserved motifs in PPOs are the HCAYC motif around CuA, the HxxxH motif around CuB, as well as the seventh, conserved, non-copper coordinating histidine (H285 in the pro-enzyme of *cg*AUS1, see Fig. 2) around CuB and the KFDV motif in the C-terminal region [5]. The coordinating histidines around CuA in *cg*AUS1, shown in Fig. 2, are H93, H116 and H125. The second histidine (H116) builds a thioether bond with the highly conserved cysteine (C97) of the H^93^CAYC^97^ motif. This thioether-bridge was also found in both catechol oxidases of *Ipomoea batatas* (*ib*CO) (see Fig. 2) [15]. Gerdemann et al. [15] proposed a possible involvement of this bridge in the catalytic pathway, but a structural function was also under discussion [15]. In *ib*CO a phenylalanine residue (F265 or F261, see Fig. 2) is in the primary sequence region of CuB, however the crystal structure shows that the bulky side chain of this residue is positioned atop the CuA site [16]. This residue, which is also present in the amino acid sequence of *cg*AUS1 (F273, in the pro-enzyme, Fig. 2) [5], was described as being responsible for preventing substrate binding and therefore hindering the monophenolase activity [16–19]. A comparable thioether-bridge building cysteine (C97 in *cg*AUS1) is present in all plant PPOs (catechol oxidases and tyrosinase). The bulky, monophenolase-blocking phenylalanine (F273 in *cg*AUS1) is present in most plant PPOs, but in some species leucine is at this position instead of phenylalanine, such as pineapple PPO from *Ananas comosus* and bread wheat PPO from *Triticum aestivum* [20]. However, these structurally relevant residues are not found in human and fungal tyrosinases as in *Agaricus bisporus* or *Neurospora crassa* and the fungal catechol oxidase from *Aspergillus oryzae*, but a thioether-forming cysteine is found in fungal tyrosinases at a different sequence location than in plant PPOs (as CXH in *ab*PPO4 and *ab*PPO3; and C(X)_16-19_H in plant PPOs, see Fig. 2) [8,20–30].

Recently, aurone synthase from *C.* *grandiflora* (*cg*AUS1) was successfully heterologously expressed in *Escherichia coli*, but recombinant aureusidin synthase (AmAS1) has not been described so far [5,8]. Further, plant catechol oxidases as PPO-6 from *Taraxacum officinale* (see Fig. 2) [27], *Malus pumila* [31] and *Physcomitrella patens* [32] have been successfully expressed in *E. coli*. PPOs from *Solanum melongena* [33] and *Camellia sinensis* [34] were expressed in inclusion bodies and refolded, but not further purified. Active tyrosinase of *Trifolium pratense* was expressed in *E. coli* as well as in transgenic *Medicago sativa* [35]. A few fungal PPOs have been cloned and heterologously expressed in *E. coli* including tyrosinases from *Verrucomicrobium spinosum* [36], *A.* *oryzae* [37] and two tyrosinases from *A.* *bisporus* [23]. Recently the first fungal, extracellular catechol oxidase from *A.* *oryzae* was functional expressed and purified [38].

Mutagenesis studies of recombinant PPOs are even more rare. In plant PPOs, the first site-directed mutagenesis study was performed of dandelion PPO-6 from *T.* *officinale* [27]. In this study Dirks-Hofmeister et al. [27] analyzed a tetrameric PPO isoenzyme (PPO-6) from dandelion (*T.* *officinale*) heterologously expressed in *E. coli* and identified, through molecular modeling, a surface-exposed cysteine (C197 in the pro-enzyme see Fig. 2, which is not existing in *cg*AUS1). Site-directed mutagenesis of this cystein to a serine proved this amino acid residue to stabilize this tetramer via a disulfide linkage [27]. The serine mutant still formed a tetrameric structure but showed impaired enzymatic efficiency and cooperativity and a reduction in stability [27]. Site-directed mutagenesis on all copper coordinating histidines, the seventh, conserved non-coordinating histidine in CuB and the thioether-bridge building cysteine was performed on fungal tyrosinase from (*ao*TYR) *A.* *oryzae* [39]. Replacements of these amino acids with asparagine resulted in mutated enzymes exhibiting no activity and containing only one copper ion per molecule tyrosinase, indicating that these residues are essential for copper incorporation and activity. Investigations on the ratio of monophenolase/diphenolase activity of tyrosinase (*bm*TYR) of *Bacillus megaterium* were performed by Goldfeder et al. [19]. They suggested that the less bulky valine (V218), which does not occur in catechol oxidases, aureusidin synthase or aurone synthase [19], allows the hydroxylation of monophenols at CuA. Therefore, Goldfeder et al. [19] exchanged V218 with a phenylalanine (V218F, which corresponds to F273 in *cg*AUS1), expecting to have a decreased monophenolase activity. However, the monophenolase activity increased surprisingly [19].

**Fig. 3:**
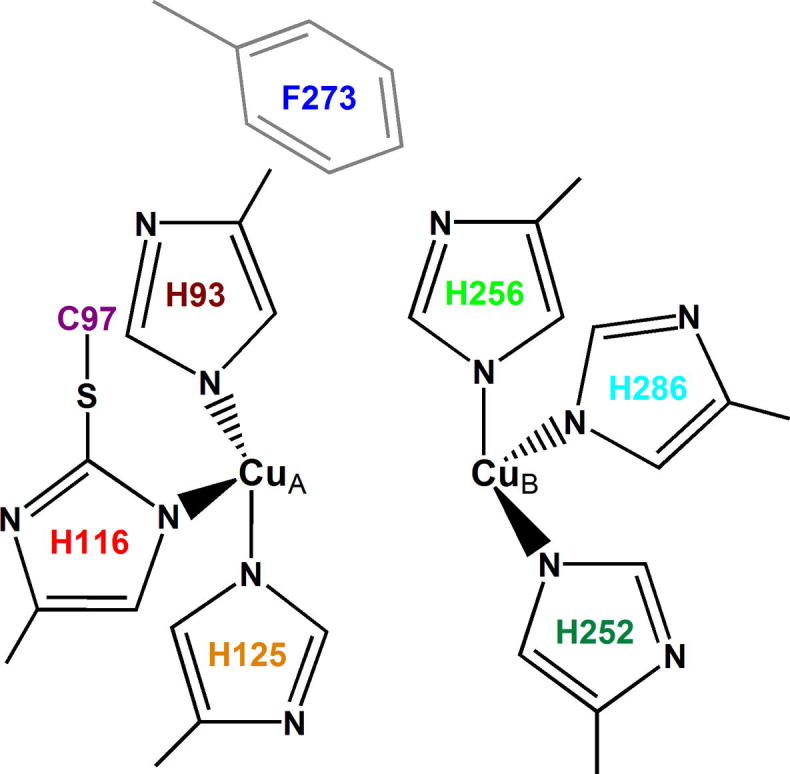
Schematic illustration of the type-3 copper center of *cg*AUS1.

The full-length sequence of *cg*AUS1 was previously described and the pro-enzyme was cloned in *E. coli*, thereby producing large amounts of pure, recombinant *cg*AUS1 [5]. *cg*AUS1 was isolated and purified from natural source as well and characterized and compared with the recombinant enzyme [40]. In order to investigate the structure and function deciding amino acids around CuA, this work reports on the first site-directed mutagenesis of *cg*AUS1 [5]. To demonstrate if all the three copper-coordinating histidines (H93, H116, H125 marked in dark red, red and orange, respectively in Fig. 3) are necessary for copper incorporation and folding, we mutated each histidine to alanine, as alanine is a non-polar amino acid, with a neutral, small side chain. Mutation of the phenylalanine (F273, marked in blue in Fig. 3) to alanine should clarify if the side chain of phenylalanine functions as a blocker residue in *cg*AUS1 and therefore influences the monophenolase/diphenolase ratio [18,19,41]. It has been suggested that the cysteine residue (C97, marked in violet in Fig. 3) forms a thioether bond with H116 [5,16]. Crystal structure investigations of *ib*CO revealed that the formed covalent bond increases structural restraints on the ligand sphere of the CuA center, therefore this cysteine (C97) was mutated to alanine in *cg*AUS1 [16]. However, Gerdemann et al. [15] supposed that this thioether-bridge does not seem to be essential, as it is absent from arthropodan HC crystal structures. Thus, a comprehensive investigation around the CuA site of aurone synthase from *C.* *grandiflora* (*cg*AUS1) is presented in this work. In order to investigate the activity of all coordinating histidines, the three histidines of CuB were mutated to alanine.

## 2 Experimental procedure

### 2.1 Cloning and site-directed mutagenesis of *cg*AUS1

Isolation and cloning of *aurone synthase* (*cgAUS1*) gene from *C. grandiflora* into pTrcHis2 vector was previously described [5]. In order to simplify protein purification the gene was, in this work, recloned into pGEX-6P-1 (GE Healthcare, Munich, Germany), which contains a GST-tag, using BamHI and EcoRI as restriction sites and including the stop codon using sticky end PCR according to Walker et al. [42]. Primers used are listed in Table 1. Site-directed mutagenesis was performed using the Q5® Site-Directed Mutagenesis Kit according to the manufacturer manual. Plasmids were transformed into NEB 5-alpha competent cells and were sequenced by a commercial supplier.

### 2.2 Heterologous expression of recombinant *cg*AUS1 in *E. coli*

Production of wild type and mutated *cg*AUS1 enzymes was performed by inoculating with overnight culture, growing at 37 °C in SB culture medium (3.2% tryptone, 2% yeast extract, 0.5% NaCl, supplemented with 100 μg/ml ampicillin), inducing with isopropyl-β-d-thiogalactopyranosid (IPTG) at an OD_600_ of 0.6 and harvesting the cells at an OD_600_ of ∼1.5, as described previously, [5]. The harvested cells were washed three times in 30 mM Tris–HCl, pH 8.5 by centrifugation at 10 000×*g* for 10 min and resuspended again in 10 ml 30 mM Tris–HCl, pH 8.5. The cells were lysed by three freeze–thaw cycles and by adding 0.3 mg/ml lysozyme (L6876, Sigma-Aldrich) and 1 mM phenylmethanesulfonylfluoride (PMSF). After adding 0.05 mg/ml DNaseI (SigmaAldrich) and 10 mM MgCl_2_ the lysate was centrifuged (25 000×*g*, 30 min, 4 °C) and directly loaded onto GSTrap FF.

### 2.3 Purification of recombinant *cg*AUS1 in *E. coli*

The GST-tagged *cg*AUS1 pro-enzymes (wild type and mutants) were purified by applying the lysate to 5 ml GSTrap FF column (GE Healthcare, Munich, Germany). All proteins were loaded onto the column using 30 mM Tris, 150 mM NaCl at pH 8.0 as binding buffer. *cg*AUS1 was eluted from the column using the same buffer supplemented with 10 mM reduced glutathione. The eluted peak was collected and buffer exchange to the binding buffer, to remove the glutathione, was performed with Vivaspin 20 30.000 MWCO PES (poly(oxy-1,4-phenylsulfonyl-1,4-phenyl)) (Sartorius Stedim Biotech GmbH, Göttingen, Germany). The GST-tag was cleaved by PreScission Protease (GE Healthcare, Munich, Germany) at 4 °C over night. The protein solution containing the GST-tag and the untagged, pure protein was loaded again onto the GSTrap FF column. GST-tag was bound onto the column and the untagged protein was eluted. The pure, untagged protein was concentrated with Vivaspin 20 30.000 MWCO PES and stored at 4 °C in 10 mM sodium acetate (pH 5.0) for further use.

### 2.4 Mono- and diphenolase activity of *cg*AUS1

The activity of the latent *cg*AUS1 enzymes was measured after cell lysis and monitored by spectrophotometric measurements (SHIMADZU UV-1800). Monophenolase activity was determined by the use of 1 ml of 10 mM sodium phosphate buffer, pH 6.5, 2.5 mM SDS as an activating agent, 33 μM l-tyrosine as substrate and up to 5 μL of enzyme solution (protein concentrations of the enzyme solutions are shown in Table 2). The increase of the absorbance at 305 nm was measured in a 1 cm quartz cuvette [43,44]. Diphenolase activity was determined with 150 μM butein, fisetin and TBC as substrate in a 125 mM sodium citrate buffer, pH 5.5, containing 2.5 mM SDS and 1 μg of enzyme solution (concentrations of *cg*AUS1 were determined by integrating the area of the collected peak after affinity chromatography, Fig. S1). Measurement was performed at 415 nm, 280 nm and 400 nm for butein, fisetin and TBC (4-tert-butylcatechol), respectively, in a 1 cm quartz cuvette [5,11].

### 2.5 Denaturing and partially denaturing SDS–PAGE analysis of *cg*AUS1

Electrophoresis was performed by the method of Laemmli [45]. The 8% polyacrylamide gels were run in a Mini-PROTEAN Tetra Cell System (BioRad, Vienna, Austria) at a constant current of 120 mV. 2 μg of purified *cg*AUS1 (including the GST-tag) were mixed with reduced loading buffer and heated for 10 min before loading to the gel.

In-gel AUS activity was determined by applying partially 8% SDS–PAGE according to Cabanes et al. [46]. The samples were mixed with loading buffer containing no β-mercaptoethanol and loaded to the gel without heating. The gel was soaked in 125 mM sodium citrate pH 5.4 containing 41 μg/ml butein. The formation of sulfuretin, as shown in Fig. 1, was monitored by a Typhoon 8600 (GE Healthcare, Munich, Germany) in the fluorescence mode using green laser (532 nm) for excitation and 555BP20 as emission filter.

### 2.6 Chymotryptic digestion and peptide mass fingerprint of *cg*AUS1 mutants

2 μg of purified protein were applied to SDS–PAGE and visualized by coomassie staining followed by excision of gel bands corresponding to the protein. After band excision destaining, washing, reduction and carbamidomethylation of cysteines was performed. In-gel digestion was achieved using chymotrypsin at 37 °C over night. Peptides were recovered from the gel using ultrasonication and dried completely by vacuum centrifugation. The samples were stored at −20 °C prior to LC–MS/MS analysis. Dried peptide samples were dissolved in 5 μL 30% formic acid (Fluka) and diluted with 40 μL eluent A (97.9% H_2_O, 2% acetonitrile, 0.1% formic acid).

Analysis of the peptide samples was carried out on a nanoUHPLC–ESI-MS/MS system using a high resolution orbitrap mass spectrometer (Dionex Ultimate 3000 RSLCnano, LTQ Velos orbitrap, Thermo Scientific). Data analysis was performed via Proteome Discoverer 1.4 by searching against the corresponding mutant sequences using Sequest as search engine. Peptide mass tolerance was 5 ppm and the fragment mass tolerance 0.5 Da. Modifications applied for each search were carbamidomethylation as fixed modification for cysteines and methionine oxidation as variable modification. For high confidence of the MS data, the false discovery rate (FDR) of the peptide spectrum matches (PSM) was set to <0.01 (Proteome Discoverer). Details about applied parameters can be found in Tables S1 and S2.

### 2.7 Quantification of copper content

Pure *cg*AUS1 wild type and mutants (Table 2) were used to determine the copper content by atom absorption spectroscopy (AAS). 3 ml of the aqueous protein solutions (concentrations are listed in Table 2) were acidified with 1 ml of 69% HNO_3_ (TraceSELECT®, Fluka, Sigma–Aldrich, Vienna, Austria) and digested for one hour at 100 °C in the oven. After cooling to room temperature the loss of liquid was filled up with deionized water. Samples were then measured directly with F-AAS (AAnalyst 300 by Perkin-Elmer, Brunn am Gebirge, Austria). Samples containing less than 0.2 mg/L Cu were measured by means of GF-AAS (PinAAcle 900Z by Perkin-Elmer, Brunn am Gebirge, Austria) using a Pd-Mg modifier. For both methods, element concentrations of each sample were calculated from the corresponding regression lines (correlation factor > 0.9995) using five different dilutions of a standard solution (Fluka, Sigma–Aldrich, Vienna, Austria). The range of calibration was 0.2–5.0 mg/L for F-AAS and 1.00–50.0 μg/L for GF-AAS, respectively.

## 3 Results and discussion

### 3.1 Heterologous expression and yield of *cg*AUS1 mutants and wild type

As a result of recloning *cg*AUS1 proenzyme into pGEX-6P-1 the recently developed method could be advanced to successfully produce large amounts of pure, active and soluble *cg*AUS1 wild type (58 kDa + 26 kDa GST tag) in *E. coli* and simplify the purification process by a reduction of the four ion exchange purification steps to only one affinity chromatography step [5]. Purification was performed under non-denaturating conditions using GSTrap FF affinity chromatography. An exemplary chromatogram of *cg*AUS1 wild type is shown in Fig. S1, where *cg*AUS1 was eluted by the use of reduced glutathione. All *cg*AUS1 mutants (H93A, H116A, H125A, H252A, H256A, H286A C97A and F273A) were expressed and purified under identical conditions, using wild type *cg*AUS1 as control sample. The amount of soluble, expressed and purified enzyme, shown in Table 2, revealed that all mutants, except the C97A mutant, showed acceptable concentrations in comparison with the wild type *cg*AUS1. The C97A mutant showed almost a less than one-tenth ratio (0.06 mg/ml) followed by F273A (0.27 mg/ml), H286A (0.33 mg/ml), H93A (0.37 mg/ml), H125A (0.74 mg/ml), H116A (1.09 mg/ml), H256A (1.20 mg/ml) and H252A (1.24 mg/ml) compared to *cg*AUS1 wild type (0.63 mg/ml). The significantly lower concentration of C97A may be due to the missing thioether bond, between C97 and H116. In contrast, Gerdemann et al. [15] proposed that this cysteine–histidine thioether bond seems not to be essential, as it is absent in arthropodan HC crystal structures. However, we suppose that this bond seems to be structurally important during expression and accurate folding. Furthermore, a thioether bond is present in all so far determined crystal structures of the type 3 copper proteins catechol oxidase, tyrosinase and molluscan hemocyanin and all of these proteins have in common two major domains (a N-terminal core domain containing the copper center and a C-terminal domain which shields the active site) [47]. In all of these structures the core domain showed a similar fold. Arthropod hemocyanin consists of three different domains (a N-terminal domain shielding the entrance of the active site, a central domain containing the active site and a immunoglobulin-like C-terminal domain). This significant structural difference might be a reason for the absence of the thioether bond in arthropod hemocyanins [48].

### 3.2 Copper incorporation

The purified *cg*AUS1 samples were used to determine the incorporated copper content by AAS (Table 2). The copper content of the wild type *cg*AUS1 was determined as being approx. 2 Cu per molecule *cg*AUS1, as expected. All three histidine mutants of CuA (H93A, H116A, H125A) contained only 1 Cu per molecule enzyme. For the F273A mutant, 2 Cu per molecule *cg*AUS1 could be determined, as it is the case in the wild type. The C97A mutant contained as well two Cu per molecule *cg*AUS1.

**Fig. 4:**
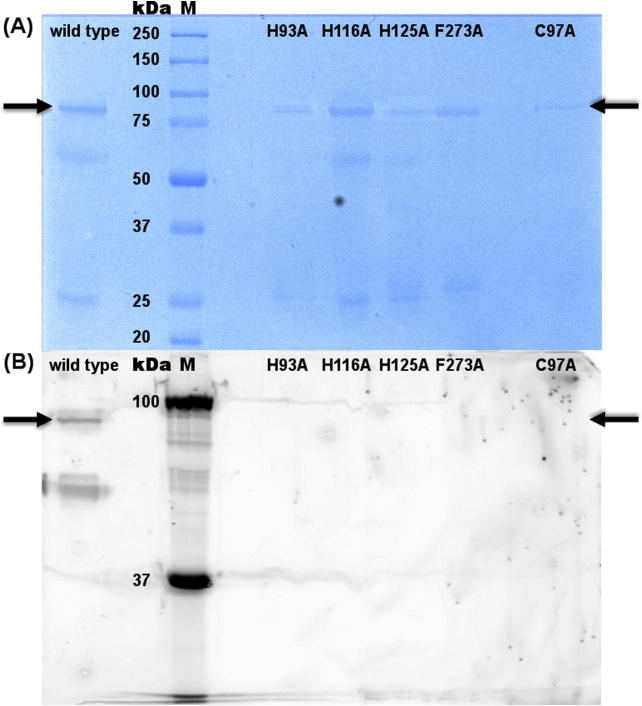
SDS–PAGE of *cg*AUS1 wild type and mutants, after purification through GSTrap FF. (A) Gel was stained with Coomassie. (B) Gel was soaked with butein. The arrows indicate the calculated size of 84 kDa, corresponding to *cg*AUS1 pro-enzyme (58 kDa) including the GST-tag (26 kDa).

In *ao*TYR the substitution of cysteine (C82) with alanine as well as substitution of the three copper coordinating histidines (H63, H84, H93) in the CuA site decreased copper binding to 50%, indicating that these mutants contain only 1 Cu per molecule [39]. This is in contrast to *cg*AUS1, where mutation of the cysteine residue did not decrease the copper content, indicating that the thioether bond seems to be structurally important for expression and folding, as well as for enzymatic catalysis (Fig. 4 and Table 3), but not essential for copper incorporation. The three copper coordinating histidines in *ao*TYR were confirmed as essential residues for copper binding and catalysis, due to the decrease of the copper content to one copper atom and the loss of mono- and diphenolase activity with l-tyrosine and l-dopa, respectively [39]. In fact, the decrease of copper content in the histidine mutants (H93A, H116A, H125A) confirms that these residues are essential for copper binding in *cg*AUS1.

### 3.3 Verification of *cg*AUS1 wild type and mutants

**Fig. 5:**
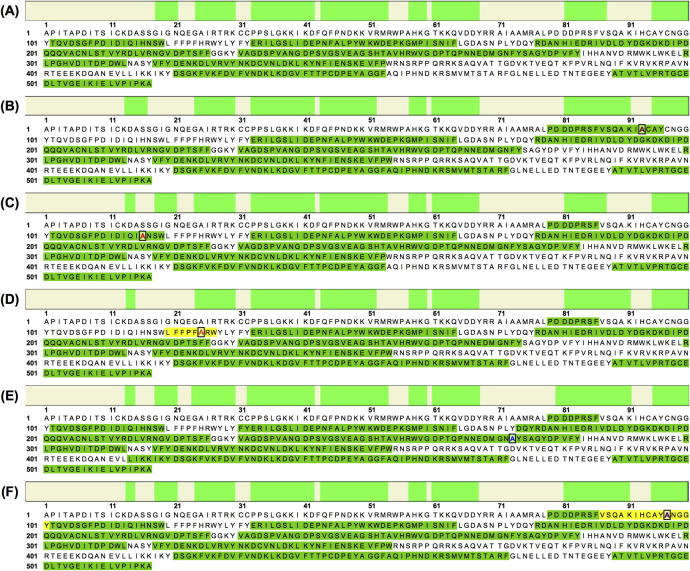
Identified peptide regions/peptides (in green) of *cg*AUS1 wild type and mutants determined by nanoUHPLC–ESI-MS/MS, including the mutated amino acids framed and colored. (A) *cg*AUS1 wild type (B) *cg*AUS1 H93A mutant (C) *cg*AUS1 H116A mutant (D) *cg*AUS1 H125A mutant, identified peak matching the peptide which contains the mutation is marked in yellow (E) *cg*AUS1 F273A mutant (F) *cg*AUS1 C97A, identified peak matching the peptide which contains the mutation is marked in yellow.

The mass of the purified *cg*AUS1 wild type and mutants were determined by SDS–PAGE. Fig. 4(A) shows samples under reduced conditions, stained with Coomassie. All samples showed a band at 84 kDa, corresponding to the calculated size of *cg*AUS1 pro-enzyme including a 26 kDa GST-tag. The smaller bands with sizes of 58 kDa and 26 kDa correlate to the *cg*AUS1 pro-enzyme without the GST-tag and the GST-tag itself, due to digestion during the cell lysis, in spite of the use of the protease inhibitor PMSF. These 84 kDa bands were excised and sequenced by nanoUHPLC–ESI-MS/MS and the identified peptides were summarized in Fig. 5 (lists of all peptides for each enzyme are shown in Tables S3–S8). Mass spectrometry sequence analysis demonstrated that all mutated *cg*AUS1 enzymes could be established. The sequence coverage was 51%, 53%, 53%, 51%, 59% and 57% for *cg*AUS1 wild type, H93A, H116A, H125A, F273A and C97A, respectively. For the mutants H93A (bold and dark red in Fig. 5(B)), H116A (bold and red in Fig. 5(C)) and F273A (bold and blue in Fig. 5(E)) peptides in the region of the mutated amino acids could be identified, verifying that the expressed protein contains the desired mutation. In contrast, the two H125A and the C97A mutants (in Fig. 5(D) and (F), respectively) showed an identical peptide pattern as *cg*AUS1 enzyme. A peptide containing the mutation site could not be determined, neither in the wild type enzyme nor in any other mutated enzyme. To further proof, signals on the MS level matching the *m/z* values of mutation-specific peptides were identified. Mutant C97A was identified, by a peak at 19.97 min with *m/z* 819.8839, matching the peptide VSQAKIHCAY**A^97^**NGGY (*m/z* 819.8883 at charge + 2, marked in yellow in Fig. 5(F)) and H125A showed a peak at 42.16 min with *m/z* 542.2969 matching the peptide LFFPF**A^116^**RW (*m/z* 542.2923 at charge + 2, marked in yellow in Fig. 5(D)). The absence of a peptide containing the third histidine of CuA is probably due to a low grade of ionization, as described previously [5,29]. All mutations were, however, verified by DNA sequencing, just as reported for tyrosinase mutants from *A.* *oryzae*, tyrosinase from *Bacillus megaterium* or PPO-6 from *T.* *officinale* [19,27,39].

### 3.4 Activity of *cg*AUS1 wild type and mutants

Fig. 4(B) shows SDS–PAGE of non-reduced samples, soaked with butein, to detect the formation of sulfuretin, see Fig. 1. Table 3 shows AUS1 activity of purified *cg*AUS1 (see also Fig. S2). The wild type *cg*AUS1 was expressed as soluble and functional enzyme, showing diphenolase activity, due to the presence of SDS as activating agent for latent enzymes in the gel [46]. All mutants of *cg*AUS1 showed no diphenolase activity with any dihydroxylated substrates such as butein, fisetin or TBC [5]. Monophenolase activity could not be observed neither for the wild type, which was expected, nor for any mutant.

The bulky CuA-site blocking phenylalanine residue (F273) was assumed to hinder monophenolated substrates in catechol oxidases [16–18]. Mauracher et al. [18] compared the crystal structure of *ib*CO with the crystal structure of tyrosinase (*ab*PPO4) from *A.* *bisporus* [49]. A superimposition of the active sites showed that, an alanine residue is located in *ab*PPO4 at the position of the phenylalanine in *ib*CO, which endorsed the assumption of a blocker residue [18]. In contrast, the lack of monophenolase and diphenolase activity of the F273A mutant in *cg*AUS1 disproves this role for the phenylalanine, as blocking residue for monophenolated substrates. This indicates, that phenylalanine 273 plays a role in substrate binding. Investigation on a less bulky valine, of *bm*TYR, at the same position of the phenylalanine showed an increase of the monophenolase activity after mutation of this valine to a phenylalanine [19]. Thus, the analysis of this *bm*TYR mutant does as well contradict a previously suggested proposal of phenylalanine as a general blocking residue in PPOs, which would eliminate the ability of the PPO to perform hydroxylation of monophenols [19,41].

In *ao*TYR site-directed mutagenesis of all copper coordinating histidines to asparagine, a single-essential cysteine residue to alanine (equivalent to C97 in *cg*AUS1) and the non-coordinating histidine in CuB as well to asparagine showed no detectable activity after mutation [39]. Nakamura et al. [39] indicated that these residues (H63, H84, H93, H290, H294, H332, H333, C82) are essential for activity, which corresponds to the results obtained with *cg*AUS1. Furthermore, both single mutations (the C97A and the H116A) demonstrated in *cg*AUS1 that this thioether bond is essential for the diphenolase activity [47,48].

## 4 Conclusion

A summary of the presented results is shown in Table 4. All selected amino acids (H93, H116, H125, F273, C97, H252, H256, H286) are essential for the full function of the enzyme and therefore essential for substrate binding. Besides the crucial presence of the histidines for Cu coordination, the F273 and C97 are presumably essential either for the substrate to bind at all, or to bind in the proper orientation for the reaction. Moreover the amino acid C97, forming a thioether bond with H116, seems to be necessary for expression as soluble enzyme in *E. coli*. We propose *in vivo* a twofold importance for C97: for proper folding of the protein structure and for diphenolase activity. The decrease of the copper content in the histidine mutants (H93A, H116A, H125) was expected, but surprisingly the loss of one copper coordinating histidine had no influence on the expression as soluble protein. However, we demonstrate that ligation of the copper at CuA site is only mediated by the three histidine residues (H93A, H116A, H125A), but is not influenced by the thioether bond. The assumption that the F273 residue acts as a blocker residue for substrates or hinders the monophenolase activity could be disproved for *cg*AUS1, as it still contains two copper atoms per molecule but lost its total activity [18,41]. We propose for *cg*AUS1 that the amino acid F273 is essential for substrate binding, but is not able to change the reaction mechanism to a tyrosinase-like enzyme.

## Conflict of interest

The authors declare no competing financial interest.

## Figures and Tables

**Table 1:** Primers used for recloning into pGEX-6P-1 and site-directed mutagenesis.

**Table 2:** Copper content of *cg*AUS1 wild type and mutants determined by atomic absorption spectroscopy (AAS). Protein concentration in mg/ml of *cg*AUS1 was determined after purification by UV at 280 nm; molecular weight (MW) and extinction coefficient for the protein concentration in μM was calculated by ProtParam [50]; copper concentration was determined by AAS; Copper/*cg*AUS1 is the determined ratio of copper per molecule AUS.

Sample	Protein [mg/ml]	MW [g/mole]	Protein [μM]	Copper [μM]	Copper/*cg*AUS1
AUS1 w/t	0.63	85 761	7.39	15.42	2.1
AUS1 H93A	0.37	85 694	4.36	4.56	1.0
AUS1 H116A	1.09	85 694	12.67	14.16	1.1
AUS1 H125A	0.74	85 694	8.65	8.97	1.0
AUS1 F273A	0.27	85 684	3.17	7.40	2.3
AUS1 C97A	0.06	85 728	0.67	1.38	2.0

**Table 3:** Diphenolase activity of *cg*AUS1 wild type and mutants determined by spectrophotometric measurements [40]. Values are calculated based on three independent measurements.

Sample	Enzymatic activity [μmol/(l∗min)]		
	Butein	Fisetin	TBC
w/t	11055	22137	125533
H93A	62	92	218
H116A	0	0	0
H125A	0	0	0
F273A	4	0	0
C97A	0	0	16
H252A	0	0	0
H256A	27	0	0
H286A	0	0	0

**Table 4:** Summary of the results obtained with various mutants in comparison to the wild type enzyme. Yield is the protein content after expression and purification; activity was determined by observing the formation of sulfuretin from butein by spectrophotometric measurements; copper incorporation was determined by AAS.

	Feature	Yield [%]	Diphenolase activity [%]	Copper incorporation [%]
*w*/*t*	–	100	100.0	100
H93A	Copper coordinating	59	0.6	50
H116A	Copper coordinating	171	0.0	54
H125A	Copper coordinating	117	0.0	50
F273A	Blocker residue atop the CuA site	43	0.0	112
C97A	Thioether bridge with H116	9	0.0	98
