# Supplementary material for: Site-directed mutagenesis around the CuA site of a polyphenol oxidase from Coreopsis grandiflora (cgAUS1)
Source: FEBS Lett. 2015 Mar 24;589(7):789–97. doi: 10.1016/j.febslet.2015.02.009 (PMC4364613; doi:10.1016/j.febslet.2015.02.009)

Site-directed mutagenesis around the CuA site of a polyphenol oxidase from *Coreopsis grandiflora* (*cg*AUS1)

Cornelia Kaintz[1], Rupert L. Mayer[2], Franz Jirsa[3], Heidi Halbwirth[4], Annette Rompel[1]

[1]Universität Wien, Fakultät für Chemie, Institut für Biophysikalische Chemie, Althanstraße 14, 1090 Wien, Österreich

[2]Universität Wien, Department of Analytical Chemistry, Währinger Straße 38, 1090 Vienna, Austria

[3]Universität Wien, Department of Inorganic Chemistry, Althanstraße 14, 1090 Vienna, Austria

[4]University of Technology Vienna, Institute of Chemical Engineering, Getreidemarkt 9, 1060 Vienna, Austria

To whom correspondence should be addressed:

Annette Rompel

phone: +43 1 4277 52502

fax: +43 1 4277 852502

email: annette.rompel@univie.ac.at

http://www.bpc.univie.ac.at

Table S 1 Experimental settings for nanoUHPLC-ESI-MS/MS measurements and data evaluation. Sample: Chymotryptic digest of purified, recombinant enzymes (*cg*AUS1 wild type and mutants).

**Reduced sample ITYR; run 1 (measured by the Department of Analytical Chemistry)**	
Device and operating software used	Nano-UHPLC (*Dionex Corporation*), Chromeleon Client Version 6.80 (*Dionex Corporation*); LTQ Orbitrap Velos (*Thermo Scientific*), LTQ Tune Plus Version 2.6.0 1065 SP3 (*Thermo Scientific*)
Software used (peak list generating)	Xcalibur 2.2 SP1.48 (*Thermo Scientific*)
Acquisition parameters	MS1 scan: m/z 200 – 3000; Filling time: 500 ms with 10^6 ions; Resolution: 60.000; Fragmentation: CID with 35 eV; Peak picking: Top6 (intensity) with isolation window 3 m/z; Resolution; 7.500; Target ion previously selected for fragmentation were dynamically excluded for 60 s with relative mass window of 5 ppm.
**Search Parameters**	
Software	Proteome Discoverer 1.4
Enzyme specificity	Chymotrypsin
Number of miss cleavages permitted	2
Search Engine	Sequest HT
Fixed modifications (including residue specificity)	Carbamidomethyl (cysteine) / +57.02 Da
Variable modifications (including residue specificity)	Oxidation (methionine) / +15.99 Da
Mass tolerance for precursor ions	±5 ppm
Mass tolerance for fragment ions	±0.6 Dalton
Relaxed False Discovery Rate	0.05
Strict False Discovery Rate	0.01
Peptide result filters	Minimum confidence = high
Name of database searched	Sequence of pro-aurone synthase 1 added manually
Cut-off score/expectation value for accepting individual MS/MS spectra provided	XCorr > 2.23

Table S 2 Parameter MIAPE (Minimum Information about a Proteomics Experiment).

Classification	Definition
**1. Ion sources — 1.1 Electrospray Ionisation (ESI)**	
Supply type (static, or fed)	fed by nano-HPLC via
Interface manufacturer, model	Nanospray Flex Ion Source (Thermo Scientific)
Sprayer type, manufacturer, model	Nanospray Flex Ion Source (Thermo Scientific)
Other parameters if discriminant for the experiment	none
**2. Post source component — 3.1 Analyser**	
Ion optics, 'simple' quadrupole, hexapole, Paul trap, linear trap, magnetic sector, FT-ICR, Orbitrap: name of the analyser(s)	MS1 survey scans in Orbitrap and MS2 analysed in LTQ ion trap
Time-of-Flight drift tube: Reflectron status	no TOF used
**3. Post-source component — 3.2 Activation / dissociation**	
Instrument component where the activation /dissociation occurs	collision-induced dissociation (CID) in ion trap
Gas type (when used)	Helium
Activation / dissociation type	CID
**4. Spectrum and peak list generation and annotation — 4.1 Data acquisition**	
Software name and version	LTQ Tune Plus Version 2.6.0 1065 SP3 (Thermo Scientific) HPLC Software : Chromeleon (Dionex Corporation), Chromeleon Client Version 6.80
Acquisition parameters	The MS1 scan (m/z 400 to 1400) was acquired in the Orbitrap with 10^6 ions (maximum filling time of 500 ms) and a resolution set to 60,000. Fragmentation was performed in the LTQ iontrap by collision-induced dissociation at 35 eV collision energy selecting the 10 most intense precursor ions (top10) with an isolation window of 3 m/z units, fragments were measured with a resolution of 7,500 in the Orbitrap. Target ions previously selected for fragmentation were dynamically excluded for 180 s with a relative mass window of 5 ppm.
**4. Spectrum and peak list generation and annotation — 4.2 Data analysis**	
Software name and version	Manual data analysis: Qual Browser as part of Xcalibur 2.2 SP1.48 (Thermo Scientific) Automatic data analysis: PEAKS Studio 6.0
**4. Spectrum and peak list generation and annotation — 4.3 Resulting data**	
Location of source ('raw') and processed files	-
The chromatogram(s) for SRM data and other relevant cases	-
m/z and intensity values	see spectra
MS level	MS1 and MS2
Ion mode	positive
For MS level 2 and higher, precursor m/z and charge if known, with the full mass spectrum / peaklist containing that precursor peak, where available	see corresponding spectra

Table S 3 List of peptides found by nanoUHPLC-ESI-MS/MS protein identification experiments of purified recombinant *cg*AUS1 wild type.

start-end	sequence	modifications	XCorr	delta mass [PPM]	peptide mass $[M+H]^+$
102-119	TQVDSGFPDIDIQIHNSW		5,52	2071,97319	0,56
133-145	ERILGSLIDEPNF		4,89	1502,78435	-0,44
137-145	GSLIDEPNF		2,81	991,4727	-0,45
140-149	IDEPNFALPY		3,21	1178,5728	-0,05
150-164	WKWDEPKGmPISNIF	M9(Oxidation)	3,22	1863,90862	-0,7
150-164	WKWDEPKGMPISNIF		4,58	1847,91521	0,11
151-164	KWDEPKGMPISNIF		4,31	1661,8366	0,55
153-164	DEPKGmPISNIF	M6(Oxidation)	3,47	1363,65593	-0,27
153-164	DEPKGMPISNIF		3,2	1347,6624	0,75
177-189	RDANHIEDRIVDL		3,3	1565,80351	0,25
177-191	RDANHIEDRIVDLDY		4,12	1843,88816	-2,84
177-208	RDANHIEDRIVDLDYDGKDKDIPDQQQVAcNL	C30(Carbamidomethyl)	5,06	3768,78676	-0,43
190-208	DYDGKDKDIPDQQQVAcNL	C17(Carbamidomethyl)	3,39	2222,00425	0,52
190-212	DYDGKDKDIPDQQQVAcNLSTVY	C17(Carbamidomethyl)	4,85	2672,21555	0,38
192-208	DGKDKDIPDQQQVAcNL	C15(Carbamidomethyl)	2,96	1943,91355	0,37
192-212	DGKDKDIPDQQQVAcNLSTVY	C15(Carbamidomethyl)	4,66	2394,12412	-0,07
213-225	RDLVRNGVDPTSF		2,35	1475,76225	1,4
213-226	RDLVRNGVDPTSFF		3,45	1622,82988	0,78
231-258	VAGDSPVANGDPSVGSVEAGSHTAVHRW		5,64	2759,31168	-0,6
259-274	VGDPTQPNNEDmGNFY	M12(Oxidation)	4,16	1813,73418	0,4
259-274	VGDPTQPNNEDMGNFY		4,54	1797,74089	1,31
275-283	SAGYDPVFY		2,38	1018,4514	-0,25

300-313	RLPGHVDITDPDWL		4,23	1633,83306	-0,19
318-326	VFYDENKDL		2,89	1142,53691	0,39
320-330	YDENKDLVRVY		4,38	1413,69902	-1,34
321-330	DENKDLVRVY		3,41	1250,63786	0,22
331-342	NKDcVNLDKLKY	C4(Carbamidomethyl)	4,69	1509,77469	1,09
343-354	NFIENSKEVFPW		3,68	1509,73784	0,34
345-354	IENSKEVFPW		3,25	1248,62627	0,24
421-431	DSGKFVKFDVF		3,35	1288,65923	1,51
429-441	DVFVNDKLKDGVF		3,94	1495,77959	0,26
432-441	VNDKLKDGVF		3,67	1134,61638	0,85
442-453	TTPcDPEYAGGF	C4(Carbamidomethyl)	4,09	1314,52519	-4,22
489-502	ATVTLVPRTGcEDL	C11(Carbamidomethyl)	2,88	1531,77947	0,6
503-517	TVGEIKIELVPIPKA		3,42	1606,97942	0,94

Table S 4 List of peptides found by nanoUHPLC-ESI-MS/MS protein identification experiments of purified recombinant *cg*AUS1 H93A mutant. Peptides identifying the mutation are marked in green.

start-end	sequence	modifications	XCorr	delta mass [PPM]	peptide mass $[M+H]^+$
79-86	PDDDPRSF		2,48	948,40563	-0,18
87-96	VSQAKI**A**cAY	C8(Carbamidomethyl)	4,03	1110,56169	0,4
133-145	ERILGSLIDEPNF		4,81	1502,78508	0,05
140-149	IDEPNFALPY		2,81	1178,5728	-0,05
150-164	WKWDEPKGMPISNIF		4,26	1847,91582	0,44
151-164	KWDEPKGmPISNIF	M8(Oxidation)	2,33	1677,83091	0,18
151-164	KWDEPKGMPISNIF		3,83	1661,8344	-0,77
153-164	DEPKGmPISNIF	M6(Oxidation)	3,62	1363,65666	0,26
153-164	DEPKGMPISNIF		3,19	1347,66142	0,02
177-189	RDANHIEDRIVDL		3,33	1565,8035	0,24
177-191	RDANHIEDRIVDLDY		3,88	1843,88944	-2,14
177-208	RDANHIEDRIVDLDYDGKDKDIPDQQQVAcNL	C30(Carbamidomethyl)	3,99	3768,78896	0,16
190-208	DYDGKDKDIPDQQQVAcNL	C17(Carbamidomethyl)	3,59	2222,00498	0,85
190-212	DYDGKDKDIPDQQQVAcNLSTVY	C17(Carbamidomethyl)	4,29	2672,20731	-2,71
192-208	DGKDKDIPDQQQVAcNL	C15(Carbamidomethyl)	2,99	1943,9141	0,65
192-212	DGKDKDIPDQQQVAcNLSTVY	C15(Carbamidomethyl)	4,11	2394,12357	-0,29
213-225	RDLVRNGVDPTSF		2,74	1475,76006	-0,09
213-226	RDLVRNGVDPTSFF		3,28	1622,82927	0,41
231-258	VAGDSPVANGDPSVGSVEAGSHTAVHRW		4,44	2759,31484	0,55
231-274	VAGDSPVANGDPSVGSVEAGSHTAVHRWVGDPTQP NNEDMGNFY		3,86	4538,03676	0,6
259-274	VGDPTQPNNEDmGNFY	M12(Oxidation)	4,21	1813,7321	-0,74
259-274	VGDPTQPNNEDMGNFY		4,37	1797,73979	0,7

300-313	RLPGHVDITDPDWL		3,53	1633,83208	-0,78
318-326	VFYDENKDL		2,7	1142,53655	0,07
320-330	YDENKDLVRVY		4,23	1413,69817	-1,94
321-330	DENKDLVRVY		3,65	1250,63823	0,51
331-340	NKDcVNLDKL	C4(Carbamidomethyl)	3,52	1218,61521	0,37
331-342	NKDcVNLDKLKY	C4(Carbamidomethyl)	5,17	1509,77286	-0,12
343-354	NFIENSKEVFPW		3,75	1509,73808	0,51
345-354	IENSKEVFPW		3,08	1248,62651	0,43
421-431	DSGKFVKFDVF		3,43	1288,65837	0,85
429-441	DVFVNDKLKDGVF		4,01	1495,78044	0,83
442-453	TTPcDPEYAGGF	C4(Carbamidomethyl)	4,33	1314,52971	-0,79
432-441	VNDKLKDGVF		3,16	1134,61601	0,53
454-472	AQIPHNDKRSmVmTSTARF	M11(Oxidation)<br>M13(Oxidation)	2,48	2222,08193	0,6
489-502	ATVTLVPRTGcEDL	C11(Carbamidomethyl)	2,89	1531,77983	0,84
503-517	TVGEIKIELVPIPKA		3,56	1606,97807	0,1

Table S 5 List of peptides found by nanoUHPLC-ESI-MS/MS protein identification experiments of purified recombinant *cg*AUS1 H116A mutant. Peptides identifying the mutation are marked in green.

start-end	sequence	modifications	XCorr	delta mass [PPM]	peptide mass $[M+H]^+$
79-86	PDDDPRSF		2,53	0,85	948,4066
102-119	TQVDSGFPDIDIQI**A**NSW		6,28	0,18	2005,95061
133-145	ERILGSLIDEPNF		4,91	-0,12	1502,78484
137-145	GSLIDEPNF		2,99	0,04	991,47319
140-145	IDEPNF		2,37	-1,94	734,33415
140-149	IDEPNFALPY		3,02	0,57	1178,57353
150-164	WKWDEPKGmPISNIF	M9(Oxidation)	3,62	-0,24	1863,90947
150-164	WKWDEPKGMPISNIF		4,57	-0,35	1847,91435
151-164	KWDEPKGmPISNIF	M8(Oxidation)	3,89	-0,13	1677,83037
151-164	KWDEPKGMPISNIF		4,38	1,06	1661,83745
153-164	DEPKGmPISNIF	M6(Oxidation)	3,63	-0,01	1363,6563
153-164	DEPKGMPISNIF		3,73	0,02	1347,66142
177-189	RDANHIEDRIVDL		3,09	0,48	1565,80387
177-191	RDANHIEDRIVDLDY		3,08	-0,02	1843,89336
177-208	RDANHIEDRIVDLDYDGKDKDIPDQQQVAcNL	C30(Carbamidomethyl)	4,43	-1,14	3768,78408
190-208	DYDGKDKDIPDQQQVAcNL	C17(Carbamidomethyl)	4,06	0,35	2222,00388
190-212	DYDGKDKDIPDQQQVAcNLSTVY	C17(Carbamidomethyl)	4,5	0,22	2672,21514
192-208	DGKDKDIPDQQQVAcNL	C15(Carbamidomethyl)	2,86	1,22	1943,9152
192-212	DGKDKDIPDQQQVAcNLSTVY	C15(Carbamidomethyl)	4,85	-1,52	2394,12064
213-225	RDLVRNGVDPTSF		3,04	-0,42	1475,75957
213-226	RDLVRNGVDPTSFF		3,48	0,63	1622,82964

231-258	VAGDSPVANGDPSVGSVEAGSHTAVHRW		4,33	0,1	2759,31362
259-274	VGDPTQPNNEDmGNFY	M12(Oxidation)	3,92	0,27	1813,73393
259-274	VGDPTQPNNEDMGNFY		4,74	1,25	1797,74077
275-283	SAGYDPVFY		2,34	-1,39	1018,45024
300-313	RLPGHVDITDPDWL		4,33	-0,93	1633,83183
318-326	VFYDENKDL		2,82	0,39	1142,53691
320-330	YDENKDLVRVY		3,85	-3,32	1413,69621
321-330	DENKDLVRVY		3,64	0,32	1250,63799
331-342	NKDcVNLDKLKY	C4(Carbamidomethyl)	4,8	-0,61	1509,77213
343-354	NFIENSKEVFPW		3,94	0,1	1509,73747
345-354	IENSKEVFPW		3,26	0,43	1248,62651
421-431	DSGKFVKFDVF		3,16	1,42	1288,65911
429-441	DVFVNDKLKDGVF		3,63	-0,23	1495,77886
432-441	VNDKLKDGVF		3,71	0,42	1134,61589
442-453	TTPcDPEYAGGF	C4(Carbamidomethyl)	4,1	-2,74	1314,52715
489-502	ATVTLVPRTGcEDL	C11(Carbamidomethyl)	3,17	0,6	1531,77947
503-517	TVGEIKIELVPIPKA		3,8	0,33	1606,97844

Table S 6 List of peptides found by nanoUHPLC-ESI-MS/MS protein identification experiments of purified recombinant *cg*AUS1 H125A mutant. Peptides identifying the mutation were not found.

start-end	sequence	modifications	XCorr	delta mass [PPM]	peptide mass $[M+H]^+$
79-86	PDDDPRSF		2,45	948,40599	0,2
133-145	ERILGSLIDEPNF		4,73	1502,78423	-0,52
140-149	IDEPNFALPY		3,2	1178,5728	-0,05
150-164	WKWDEPKGMPISNIF		4,05	1847,91484	-0,09
150-164	WKWDEPKGmPISNIF	M9(Oxidation)	2,72	1863,9052	-2,53
151-164	KWDEPKGMPISNIF		4,2	1661,83562	-0,04
153-164	DEPKGMPISNIF		3,84	1347,66118	-0,16
153-164	DEPKGmPISNIF	M6(Oxidation)	3,55	1363,65483	-1,08
177-189	RDANHIEDRIVDL		3,39	1565,80315	0,02
177-191	RDANHIEDRIVDLDY		3,78	1843,88834	-2,74
177-208	RDANHIEDRIVDLDYDGKDKDIPDQQQVAcNL	C30(Carbamidomethyl)	5,34	3768,7853	-0,82
190-208	DYDGKDKDIPDQQQVAcNL	C17(Carbamidomethyl)	3,13	2222,00461	0,68
190-212	DYDGKDKDIPDQQQVAcNLSTVY	C17(Carbamidomethyl)	4,29	2672,21408	-0,17
192-208	DGKDKDIPDQQQVAcNL	C15(Carbamidomethyl)	2,77	1943,9152	1,22
192-212	DGKDKDIPDQQQVAcNLSTVY	C15(Carbamidomethyl)	3,89	2394,1232	-0,45
213-225	RDLVRNGVDPTSF		2,68	1475,76274	1,73
213-226	RDLVRNGVDPTSFF		3,08	1622,82976	0,71
231-258	VAGDSPVANGDPSVGSVEAGSHTAVHRW		4,13	2759,31557	0,81
231-274	VAGDSPVANGDPSVGSVEAGSHTAVHRWVGDPTQPNNEDmGNFY	M40(Oxidation)	4,32	4554,03139	0,54
259-274	VGDPTQPNNEDMGNFY		4,69	1797,73796	-0,32
259-274	VGDPTQPNNEDmGNFY	M12(Oxidation)	4,54	1813,73332	-0,07
300-313	RLPGHVDITDPDWL		3,72	1633,8333	-0,04

318-326	VFYDENKDL		2,89	1142,53667	0,17
320-330	YDENKDLVRVY		4,46	1413,69621	-3,32
321-330	DENKDLVRVY		3,89	1250,63835	0,61
331-340	NKDcVNLDKL	C4(Carbamidomethyl)	3,29	1218,61493	0,15
331-342	NKDcVNLDKLKY	C4(Carbamidomethyl)	4,63	1509,77369	0,42
343-354	NFIENSKEVFPW		3,43	1509,73796	0,42
345-354	IENSKEVFPW		3,1	1248,62712	0,92
421-431	DSGKFVKFDVF		3,77	1288,65715	-0,1
429-441	DVFVNDKLKDGVF		4,16	1495,77873	-0,31
432-441	VNDKLKDGVF		3,24	1134,61528	-0,11
442-453	TTPcDPEYAGGF	C4(Carbamidomethyl)	4,18	1314,52715	-2,74
454-472	AQIPHNDKRSMVmTSTARF	M13(Oxidation)	2,18	2206,0873	0,74
489-502	ATVTLVPRTGcEDL	C11(Carbamidomethyl)	2,9	1531,77971	0,76
494-517	VPRTGcEDLTVGEIKIELVPIPKA	C6(Carbamidomethyl)	3,35	2634,45424	0,23
503-517	TVGEIKIELVPIPKA		3,57	1606,97843	0,32

Table S 7 List of peptides found by nanoUHPLC-ESI-MS/MS protein identification experiments of purified recombinant *cg*AUS1 F273A mutant. Peptides identifying the mutation are marked in green.

start-end	sequence	modifications	XCorr	delta mass [PPM]	peptide mass $[M+H]^+$
79-86	PDDDPRSF		2,3	948,40599	0,2
102-119	TQVDSGFPDIDIQIHNSW		5,92	2071,97173	-0,15
131-136	FYERIL		2,15	840,46245	1,2
133-145	ERILGSLIDEPNF		4,82	1502,78447	-0,36
137-145	GSLIDEPNF		3,03	991,47301	-0,14
140-145	IDEPNF		1,95	734,3333	-3,11
140-149	IDEPNFALPY		3,34	1178,5728	-0,05
150-164	WKWDEPKGmPISNIF	M9(Oxidation)	3,83	1863,90911	-0,44
150-164	WKWDEPKGMPISNIF		4,58	1847,91252	-1,34
151-164	KWDEPKGmPISNIF	M8(Oxidation)	3,59	1677,83025	-0,21
151-164	KWDEPKGMPISNIF		4,3	1661,83476	-0,55
153-164	DEPKGmPISNIF	M6(Oxidation)	3,48	1363,6563	-0,01
153-164	DEPKGMPISNIF		3,57	1347,66216	0,57
174-189	DQYRDANHIEDRIVDL		3,83	1971,95157	-0,2
177-189	RDANHIEDRIVDL		3,2	1565,80222	-0,58
177-191	RDANHIEDRIVDLDY		3,64	1843,88651	-3,73
177-208	RDANHIEDRIVDLDYDGKDKDIPDQQQVAcNL	C30(Carbamidomethyl)	6,06	3768,78586	-0,67
190-208	DYDGKDKDIPDQQQVAcNL	C17(Carbamidomethyl)	3,25	2222,0037	0,27
190-212	DYDGKDKDIPDQQQVAcNLSTVY	C17(Carbamidomethyl)	4,42	2672,20639	-3,05
192-208	DGKDKDIPDQQQVAcNL	C15(Carbamidomethyl)	2,83	1943,91465	0,94
192-212	DGKDKDIPDQQQVAcNLSTVY	C15(Carbamidomethyl)	5,33	2394,12383	-0,19

213-225	RDLVRNGVDPTSF		2,69	1475,75969	-0,34
213-226	RDLVRNGVDPTSFF		3,37	1622,82988	0,78
213-274	VAGDSPVANGDPSVGSVEAGSHTAVHRWVGDPTQPNNEDMGN**A**Y		6,45	4462,00161	-0,25
231-258	VAGDSPVANGDPSVGSVEAGSHTAVHRW		5,88	2759,31424	0,33
259-274	VGDPTQPNNEDmGN**A**Y	M12(Oxidation)	3,84	1737,70244	0,17
259-274	VGDPTQPNNEDMGN**A**Y		3,78	1721,70647	-0,44
275-283	SAGYDPVFY		2,53	1018,45043	-1,21
300-313	RLPGHVDITDPDWL		3,92	1633,83159	-1,08
318-326	VFYDENKDL		2,94	1142,53777	1,13
320-330	YDENKDLVRVY		4,34	1413,69805	-2,03
321-330	DENKDLVRVY		3,89	1250,63762	0,02
331-342	NKDcVNLDKLKY	C4(Carbamidomethyl)	5,28	1509,77039	-1,76
343-354	NFIENSKEVFPW		4,06	1509,73711	-0,14
345-354	IENSKEVFPW		3,46	1248,62724	1,02
414-420	LIKKIKY		1,94	905,61815	-0,14
421-431	DSGKFVKFDVF		3,47	1288,65874	1,13
426-431	VKFDVF		1,96	754,41271	-0,97
429-441	DVFVNDKLKDGVF		3,94	1495,77995	0,5
432-441	VNDKLKDGVF		2,42	1134,61626	0,75
442-453	TTPcDPEYAGGF	C4(Carbamidomethyl)	3,94	1314,53154	0,61
454-472	AQIPHNDKRSmVmTSTARF	M11(Oxidation) M13(Oxidation)	2,48	2222,07949	-0,5
454-472	AQIPHNDKRSMVmTSTARF	M13(Oxidation)	2,06	2206,08413	-0,7
489-502	ATVTLVPRTGcEDL	C11(Carbamidomethyl)	2,97	1531,77886	0,2
494-517	VPRTGcEDLTVGEIKIELVPIPKA	C6(Carbamidomethyl)	5	2634,45444	0,3
503-517	TVGEIKIELVPIPKA		3,65	1606,97966	1,09

Table S 8 List of peptides found by nanoUHPLC-ESI-MS/MS protein identification experiments of purified recombinant *cg*AUS1 C97A mutant. Peptides identifying the mutation were not found.

start-end	sequence	modifications	XCorr	delta mass [PPM]	peptide mass $[M+H]^+$
79-86	PDDDPRSF		2,81	-0,44	948,40538
102-119	TQVDSGFPDIDIQIHNSW		5,65	0,08	2071,97222
133-145	ERILGSLIDEPNF		4,22	0,29	1502,78545
140-149	IDEPNFALPY		2,82	0,36	1178,57329
150-164	WKWDEPKGmPISNIF	M9(Oxidation)	3,56	-0,7	1863,90862
150-164	WKWDEPKGMPISNIF		4,62	-0,42	1847,91423
151-164	KWDEPKGMPISNIF		4,34	-0,55	1661,83476
153-164	DEPKGmPISNIF	M6(Oxidation)	3,64	0,62	1363,65715
153-164	DEPKGMPISNIF		3,58	-0,61	1347,66057
177-189	RDANHIEDRIVDL		3,31	0,13	1565,80332
177-191	RDANHIEDRIVDLDY		3,95	-1,55	1843,89054
177-208	RDANHIEDRIVDLDYDGKDKDIPDQQQVAcNL	C30(Carbamidomethyl)	4,81	2,36	3768,79726
190-208	DYDGKDKDIPDQQQVAcNL	C17(Carbamidomethyl)	3,27	0,35	2222,00388
190-212	DYDGKDKDIPDQQQVAcNLSTVY	C17(Carbamidomethyl)	4,23	-2,5	2672,20786
192-208	DGKDKDIPDQQQVAcNL	C15(Carbamidomethyl)	2,57	0,37	1943,91355
192-212	DGKDKDIPDQQQVAcNLSTVY	C15(Carbamidomethyl)	3,29	-0,07	2394,12412
213-225	RDLVRNGVDPTSF		2,15	-0,34	1475,75969
213-226	RDLVRNGVDPTSFF		3,66	0,93	1622,83013
231-258	VAGDSPVANGDPSVGSVEAGSHTAVHRW		4,24	0,13	2759,31369
259-274	VGDPTQPNNEDmGNFY	M12(Oxidation)	4,11	0,2	1813,73381
259-274	VGDPTQPNNEDMGNFY		4,51	0,29	1797,73906

275-283	SAGYDPVFY		2,2	0,17	1018,45183
300-313	RLPGHVDITDPDWL		4,24	-0,11	1633,83318
318-326	VFYDENKDL		2,55	0,6	1142,53716
320-330	YDENKDLVRVY		3,44	0,3	1413,70134
321-330	DENKDLVRVY		3,51	-0,27	1250,63725
331-340	NKDcVNLDKL	C4(Carbamidomethyl)	3,64	0,52	1218,61539
331-342	NKDcVNLDKLKY	C4(Carbamidomethyl)	5,04	0,55	1509,77387
343-354	NFIENSKEVFPW		3,99	0,51	1509,73808
345-354	IENSKEVFPW		3,04	0,04	1248,62602
421-428	DSGKFVKF		2,45	0,55	927,49401
421-431	DSGKFVKFDVF		2,9	0,09	1288,6574
429-441	DVFVNDKLKDGVF		4,03	0,5	1495,77995
432-441	VNDKLKDGVF		2,88	-0,22	1134,61516
442-453	TTPcDPEYAGGF	C4(Carbamidomethyl)	4,06	-4,13	1314,52532
454-472	AQIPHNDKRSmVmTSTARF	M11(Oxidation); M13(Oxidation)	2,57	0,93	2222,08266
454-472	AQIPHNDKRSMVmTSTARF	M13(Oxidation)	2,1	0,4	2206,08657
489-502	ATVTLVPRTGcEDL	C11(Carbamidomethyl)	2,91	-0,12	1531,77837
494-502	VPRTGcEDL	C6(Carbamidomethyl)	2,04	-0,11	1046,49346
503-517	TVGEIKIELVPIPKA		3,76	-0,13	1606,97771

Fig. S 1 Affinity chromatographic (FPLC) run of *cg*AUS1 wild type on GSTrap FF. Legend: — UV absorbance at 280 nm [mAU], — UV absorbance at 345 nm [mAU], — gradient [% buffer B].

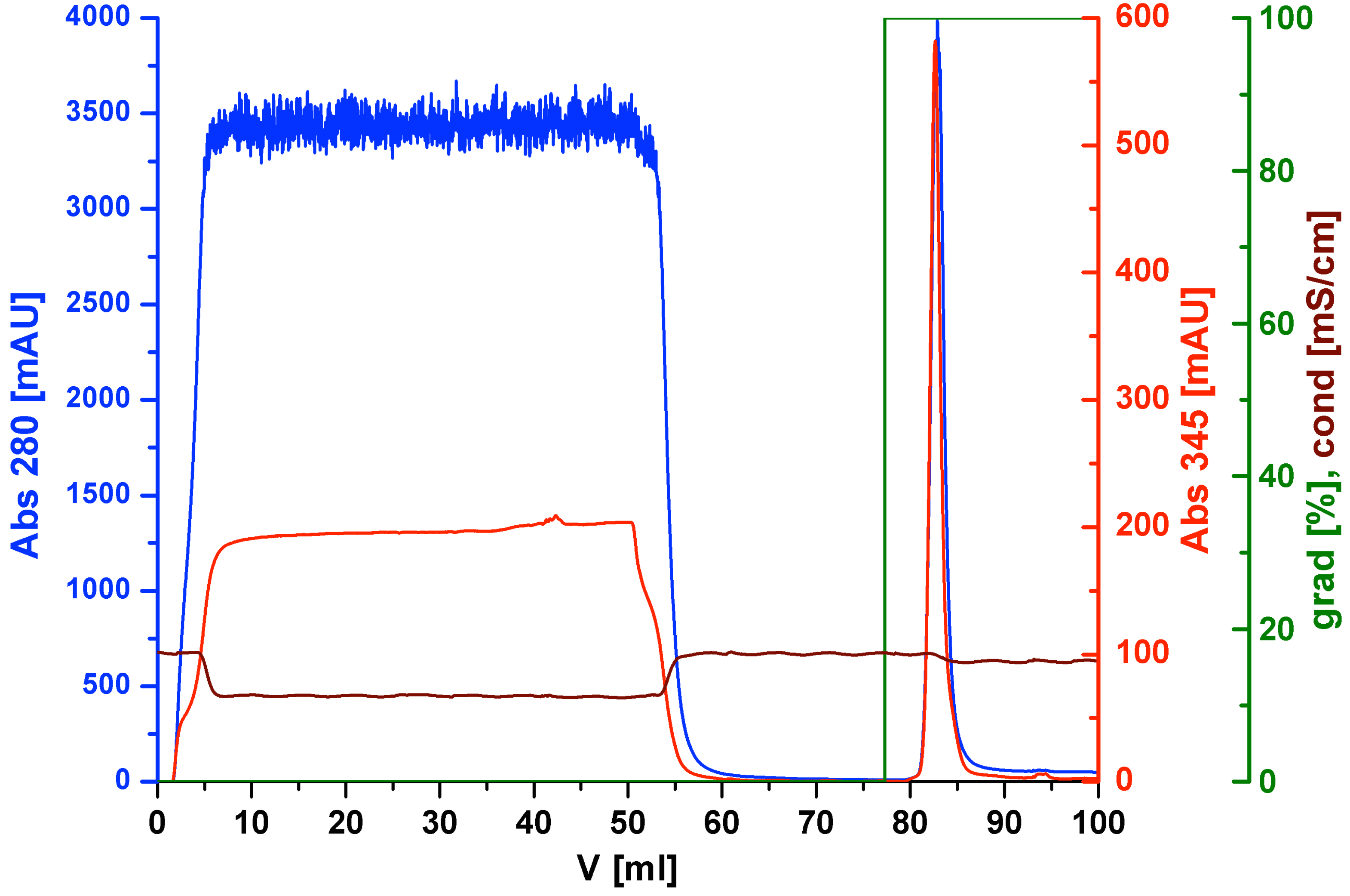

Fig. S 2 Spectrophotometric measurements of *cg*AUS1 wild type and mutants after cell lysis. (A) with butein as substrate (B) with fisetin as substrate (C) with TBC as substrate. Data shown are an average of three measurements.

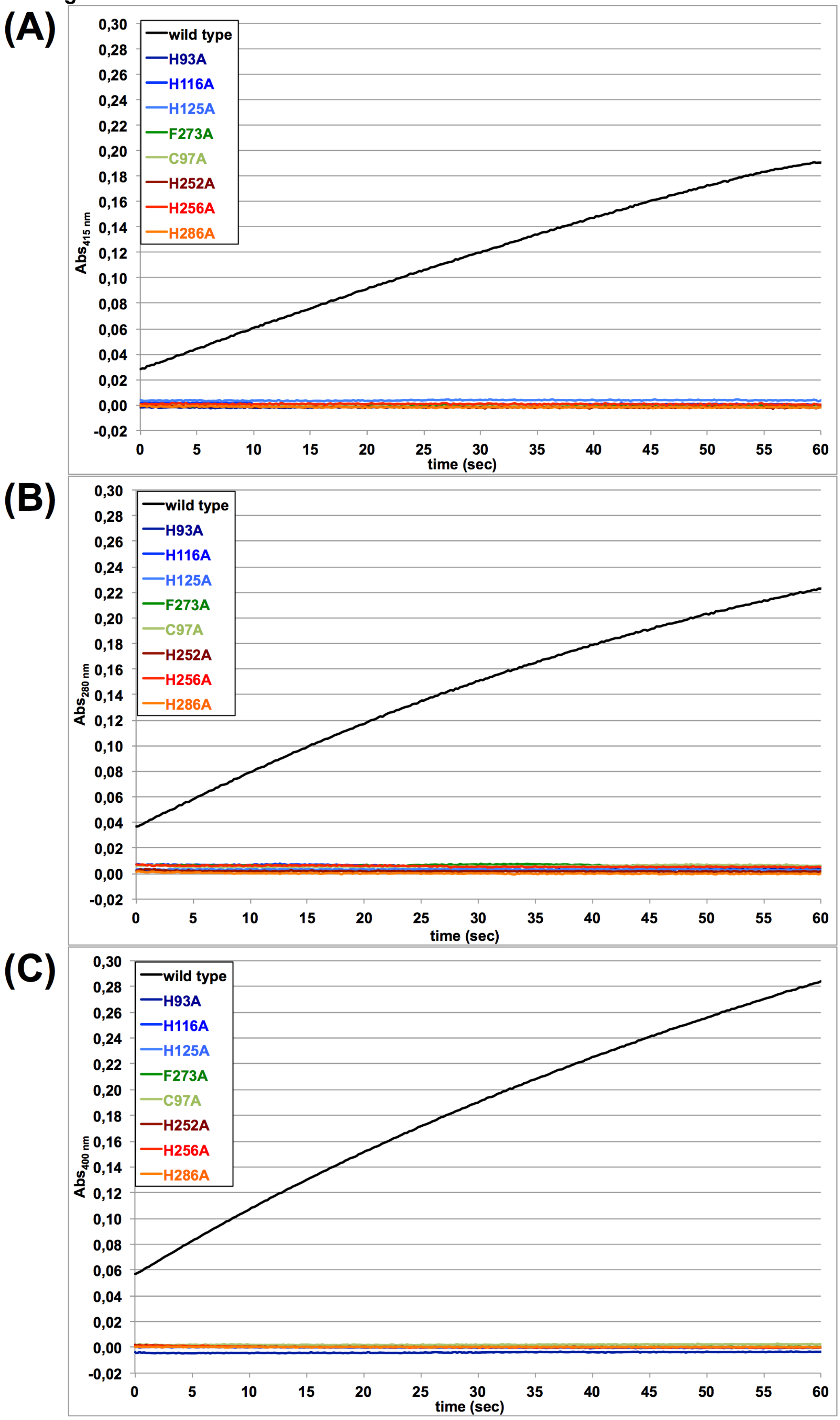

Supplement: Supplementary data 1 [file mmc1.pdf]
